# Perspectives of middle and older aged adults in primary care on acceptability of blood‐based biomarkers to identify risk of Alzheimer's disease

**DOI:** 10.1002/alz70860_103739

**Published:** 2025-12-23

**Authors:** Andrea M Russell, Jeff Williamson, Wei Huang, Rebecca Lovett, Stephanie Batio, Jeimmy Hurtado, Morgan Bonham, Michelle M Mielke, Chinedu T Udeh‐Momoh, Michael S Wolf

**Affiliations:** ^1^ Northwestern University, Chicago, IL, USA; ^2^ Wake Forest University School of Medicine, Winston‐Salem, NC, USA; ^3^ Division of Public Health Sciences, Wake Forest University, School of Medicine, Winston‐Salem, NC, USA

## Abstract

**Background:**

Blood‐based biomarkers (BBMs) for Alzheimer's Disease (AD) pathophysiology could be accessible and cost‐effective for disease detection and monitoring in primary care. Few studies have examined attitudes or acceptability toward BBMs among patients.

**Methods:**

Patients aged 30+ with primary care providers (PCPs) and participating in ongoing observational studies in Chicago IL were invited to complete a questionnaire online or by phone. Participants were provided with a brief explanation about BBMs and asked about their attitudes. Descriptive statistics summarized sample characteristics. Chi‐squares and ordinal logistic regression controlling for age and sex examined associations between demographic variables, health literacy (HL) and survey items.

**Results:**

Participants (*N* = 572) were, on average, 61.9 years old (SD 12.8), 27.8% were Black, 10.5% Hispanic, and 28.7% had limited HL. Most participants (96.6%) indicated they would complete BBMs if recommended by their PCP and 87.3% said a positive test would very likely motivate them to engage in risk reduction. About a third (35.8%) of participants had some or strong concerns about test accuracy, which varied by education (College Graduate: 40.1% vs. High School (HS) Graduate or Less: 31.7%, *p* = 0.009). Approximately a quarter (27.5%) anticipated distress upon positive result was likely or very likely, differing by education (College Graduate: 31.2% vs. HS Graduate or less: 10.5%, *p* = 0.001) and HL (Adequate: 31.5% vs Limited: 17.4%, *p* <0.001). Women reported higher concern for distress following a positive test (Women: 32.4% vs. Men: 19.3%, *p* = 0.003). Associations persisted in multivariable models, except the association between education association and accuracy concerns. Factors participants endorsed that would increase acceptability were: if results inform future care (94.6%), if tests were covered by insurance (93.9%), ease/convenience of the testing (88.3%), provision of comprehensive education before testing (88.9%), and endorsement from PCPs (82.1%). Despite concerns, most participants believed BBMs should be offered to patients with memory changes (94.6%) and those age 65+(87.2%).

**Conclusions:**

BBMs are acceptable to primary care patients. Affordability, ability to inform future care, and convenience will promote uptake in middle aged and older adults. Prior to testing, high‐quality education is recommended. Based on results, PCPs should provide resources to address potential distress and information needs.

